# Influence of Geographical Origin and Processing Methods on Biopreservatives, Heavy Metals, Lipid Quality Indices, and Aflatoxin M1 in Imported Cheeses: A Public Health Concern

**DOI:** 10.1002/fsn3.70981

**Published:** 2025-09-18

**Authors:** Mohammed A. Falih, Qausar Hamed Alkaisy, Ammar B. Altemimi, Mohammad Ali Hesarinejad, Tarek Gamal Abedelmaksoud

**Affiliations:** ^1^ Department of Dairy Science and Technology, College of Food Sciences University of AL‐Qasim Green Al Qasim Iraq; ^2^ Department of Food Science College of Agriculture, University of Basrah Basrah Iraq; ^3^ Department of Food Sensory and Cognitive Science Research Institute of Food Science and Technology (RIFST) Mashhad Iran; ^4^ Food Science Department, Faculty of Agriculture Cairo University Giza Egypt

**Keywords:** cheese, heavy metals, natamycin, nisin, soft cheese

## Abstract

In this study, some safety and quality parameters such as biopreservatives, heavy metals, lipid quality indices, and levels of aflatoxin M1 of imported cheeses in the Babylon Governorate of Iraq were examined by focusing on how processing methods and geographical origins. A total of 3 different cheese types and 27 samples from Iran, Saudi Arabia, and Turkey were analyzed by novel techniques such as High‐Performance Liquid Chromatography and Liquid Chromatography‐Mass Spectrometry. The findings showed the presence of nisin A and nisin Z presence in a few samples, while natamycin was detected in only one soft cheese in an amount below the regulatory limit. Analysis of fatty acid composition revealed high variability as well as high levels of SFA among samples from different countries. Detectable levels of cadmium, lead, copper, and arsenic were also found in some cheeses, in some cases exceeding the maximum permitted levels. Aflatoxin M1 contamination was also found in a handful of cheese samples, with several exceeding the maximum permitted levels. These findings revealed significant differences in the quality and safety of cheeses based on origin and processing methods, raising potential public health concerns related to the consumption of imported cheeses. The current study also strongly emphasized the need for improved quality control measures and regular monitoring of dairy products to ensure consumer safety and compliance with international health standards. In addition, it strongly recommends further research to better understand the long‐term effects of these pollutants on public health.

## Graphical abstract

This study evaluates the impact of geographical origin and processing methods on biopreservatives, heavy metals, lipid quality indices, and aflatoxin M1 levels in imported cheeses from Iran, Turkey, and Saudi Arabia. Findings reveal significant variations linked to production practices, highlighting potential public health risks. The research underscores the need for stricter food safety regulations to mitigate contamination in cheese products.

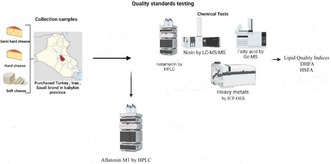

## 1 Introduction

Cheese production is a significant sector in the food industry, contributing to the provision of proteins, fats, and various other essential nutrients. As the demand for processed and packaged foods increases, various preservatives such as natamycin and nisin are used to maintain the quality and safety of these products during storage and transportation. While these preservatives help inhibit microbial growth, their use in food products requires careful monitoring, especially due to growing concerns over their potential health effects on consumers. Therefore, studying preservatives in different types of cheese is crucial to evaluating how these products comply with international health standards (Nájera et al. 2021; Fernandes et al. 2025).

In recent years, there has been increasing interest in food products produced in various countries such as Iran, Saudi Arabia, and Turkey, particularly in the cheese industry. These products exhibit diverse ingredients in terms of production and flavor, raising questions about the use of preservatives and their quality control through periodic monitoring. This study aims to provide a detailed analysis of several types of cheeses imported from these countries, focusing on the levels of preservatives such as natamycin and nisin, as well as monitoring the levels of fats, fatty acids, and heavy metals they may contain (Meena et al. 2021).

Natamycin is one of the most widely used preservatives in cheese, added to prevent the growth of yeasts and molds, thereby extending the shelf life of the product. Additionally, nisin is another natural preservative used in some cheeses as an alternative to natamycin. In this study, advanced techniques such as High‐Performance Liquid Chromatography (HPLC) and LC–MS–MS are utilized to detect these preservatives in different cheese samples. The study explores the extent to which the imported cheeses from Iran, Saudi Arabia, and Turkey meet the specified standards for food additives (Ozdemir et al. 2019; Haq et al. 2024).

In addition to focusing on preservatives, the study also aims to examine the distribution and percentages of saturated and unsaturated fatty acids in these cheeses. Fatty acids are among the key factors influencing the nutritional and health value of foods. For instance, unsaturated fatty acids, such as oleic and linoleic acids, are beneficial for health, as they contribute to lowering cholesterol levels in the blood and improving cardiovascular health. On the other hand, some cheeses contain high levels of saturated fatty acids, such as palmitic and stearic acids, which may contribute to an increased risk of cardiovascular diseases when consumed in large quantities (Paszczyk 2022; Ponnampalam et al. 2024).

Moreover, studying the levels of unsaturated and saturated fats in cheeses imported from Iran, Saudi Arabia, and Turkey helps to identify their potential impacts on public health. Additionally, the analysis of heavy metals such as lead, cadmium, copper, and mercury is an essential factor in ensuring the safety of food products. These metals can pose significant risks to consumer health if consumed in high amounts, making it necessary to determine their levels in various cheeses (Othman 2010; Muneam et al. 2023).

This study will assess the safety and nutritional quality of imported cheeses from Iran, Saudi Arabia, and Turkey by analyzing preservative levels, fatty acid distribution, and heavy metal content. Using HPLC and LC‐MS‐MS, we will evaluate natamycin and nisin levels, measure the distribution of fatty acids, and monitor heavy metals like cadmium, lead, copper, and mercury to ensure compliance with safety standards. The study will also explore differences in preservative levels and composition across the cheeses from different countries, offering health recommendations to improve food safety and raise awareness of potential risks. The main hypothesis of the current study is that the geographical origin and processing methods of imported cheeses significantly influence their levels of biopreservatives, heavy metals, lipid quality indices, and aflatoxin M1. It was predicted that differences in national food safety regulations, environmental contamination, animal feed quality, and cheese processing practices among these countries would result in evaluated variations in cheese safety and nutritional quality.

## 2 Materials and Methods

### 2.1 Samples Collections

A total of 27 different cheese samples were collected, with each sample analyzed in triplicate, resulting in a total of 81 samples covering all cheese types included in the study. The selected cheeses were sourced from various manufacturers and were commercially available in markets and retail shops across Babylon Governorate, Iraq, during the period from November 2024 to February 2025. These samples originated from three countries: Iran, Turkey, and Saudi Arabia. The cheeses were categorized into three main groups: soft cheese, semi‐hard cheese, and hard cheese.

### 2.2 Extraction and Determination of Natamycin

**FIGURE 1:**
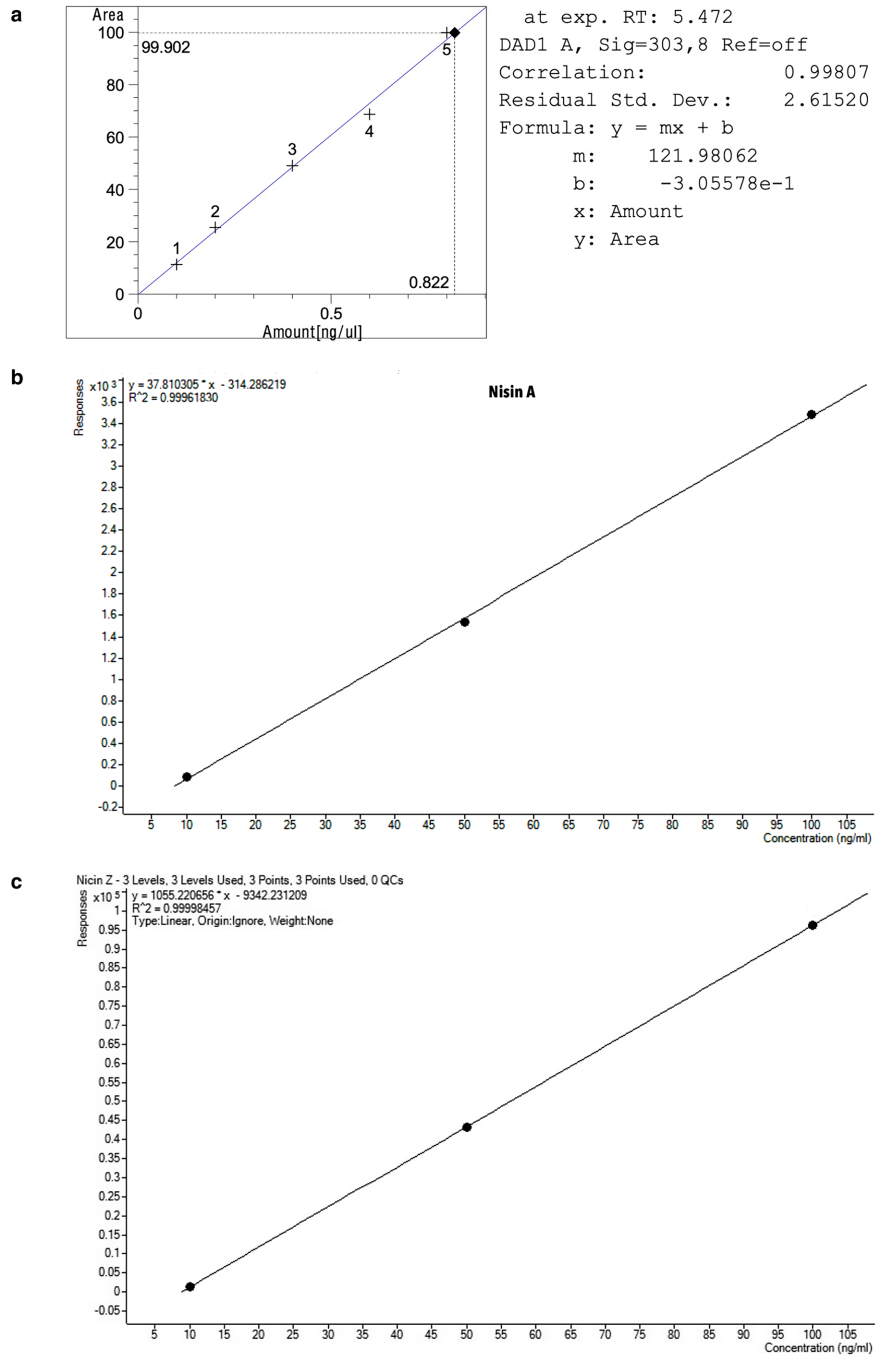
Calibration curve for standard natamycin (a), nisin A (b), and nisin Z (c).

10 g of cheese samples were weighed into a conical flask and 100 mL of methanol was added. The mixture was stirred for 90 min in a magnetic stirrer and then 50 mL of deionized water was added to this solution. It was placed in the freezer for approximately 60 min. The cold extract was filtered through filter paper (Macherey‐Nagel 100,751/60/030). The solution was left at room temperature. A portion of the filtrate was filtered through a micromembrane filter with a pore size of 0.45 μm and then 0.20 μm. The resulting filtrate of 20 μL per injection was used for direct chromatographic analysis in an HPLC (Ozdemir et al. 2019). An HPLC (Agilent 1200) consisting of a quaternary pump (G1311A), an automatic injection system (G1330B), and a vertical thermostat (G1316A) (410 × 435 × 140) was used at 25°C. A multi‐wavelength detector was used according to ISO 13829‐2. A diode array detector (G1315D) was used at 305 nm. The mobile phase was a mixture (H_2_O: methanol: acetic acid) at a ratio of (1:12:4) and a flow rate of 0.8 mL/min. A calibration curve was prepared at concentrations of (0, 0.1, 0.2, 0.4, 0.6, 0.8) ng/mL for standard antamycin Figure 1.

### 2.3 Extraction and Determination of Nisin

Two grams of cheese were weighed and placed in a 50‐mL beaker. 10 mL of 0.02 N hydrochloric acid (pH 2) was added with continuous stirring for 30 min using a stirrer. The mixture was then placed on a hot plate (preheated to 100°C) with magnetic stirring and boiled for 5 min. 40 mL of 0.02 N hydrochloric acid was added to the mixture, transferred to a 50‐ml tube, and centrifuged at 4500 rpm for 20 min at 4°C (Eppendorf 5810 R centrifuge, Hørsholm, Denmark) (Martinez‐Rios et al. 2021). The filtrate was collected for nisin concentration determination using LC–MS–MS. A Triple Quad 6420 LC–MS–MS equipped with a Dynamic and Triggered MRM system and an Electrospray Ionization (ESI) source (Agilent, Santa Clara, USA) was used, coupled to a Dionex Thermo Scientific (Waltham, MA, USA) 3000 UHPLC. The UHPLC system consists of a pump and an autosampler. Chromatographic separation was performed using a Zorbax 300 SB‐C18 column. The mobile phase was (H_2_O:MeOH (50:50) 0.1% formic acid) according to the ISIRI 13574 method developed by the Iranian Standards and Industrial Research Organization. The column temperature was maintained at 35°C, and the mobile phase flow rate was 0.3 mL/min. The multiple reaction monitoring (MRM) parameters for both nisin A and nisin Z were specified in Table 1. A calibration curve was also prepared (Figure 1) at concentrations of 10–100 ng/mL for both nisin A and nisin Z.

**TABLE 1:** Multiple reaction monitoring parameters for nisin A and nisin Z for LC–MS–MS analysis.

Collision energy (eV)	Fragmentor (V)	Fragment ion (m/z)	Parent ion (m/Z)	Nisin
10	105	810.8	671.0	A
110	120	805.1	666.9	Z

### 2.4 Fatty Acids Analysis (GC–MS)

The esterification of fatty acids was carried out by mixing 50 mg of cheese with 1 mL of n‐hexane and 0.2 mL of KOH/MeOH (0.2 M). The sample was shaken for 3 min and allowed to stand for 15 min, then 1 mL of HCl/MeOH (10%) was added. The sample was shaken for 10 s and then incubated at 50°C for 10 min. After cooling the samples, 2 mL of ultrapure water and 2 mL of n‐hexane were added. The sample was mixed for 10 s and centrifuged for 5 min, followed by 0.5 g of anhydrous sodium sulfate. The sample was then shaken for 30 s and centrifuged for 5 min until the layers separated. The upper layer represented approximately 20% of the resulting methyl esters of fatty acids, making it ready for direct injection into gas chromatography–mass spectrometry (GC–MS) (Narloch and Wejnerowska 2023). Cheese samples were analyzed using GC–MS in the laboratory of the Laboratories and Quality Control Department of Basra Oil Company, using an Agilent 7890/5977A MSD equipped with a HP‐5 ms separation column (30 m × 0.32 mm × 0.25 μm). A 1 μL sample was injected using a split‐mode injection system at 290°C, with a flow rate of 1 mL/min of high‐purity helium gas. The oven program was set at an initial temperature of 40°C for 5 min, then the temperature was gradually increased at a rate of 10°C/min until it reached 300°C. Finally, the resulting peaks were matched to the NIST14 spectral library to identify the compounds.

### 2.5 Lipid Quality Indices

The following equations were used to determine lipid quality indices based on fatty acid ratios estimated by GC–MS, according to Paszczyk et al. (2022):

$$\text{Hypocholesterolaemic fatty acids}\;\left(\text{DHFA}\right)=\sum \mathrm{UFA}+C18:0$$

$$\text{Hypercholesterolaemic fatty acids}\;\left(\text{HSFA}\right)=\sum \mathrm{SFA}-C18:0$$

$$\frac{\text{Hypocholesterolaemic}}{\text{Hypercholesterolaemic}}\text{ratio}\;\left(\frac{\mathrm{Hpo}}{\text{Hper}}\right)=\frac{\left(C18:1n\;9+C18:2n\;6+C18:3n\;3\right)}{C12:0+C14:0+C16:0}$$

### 2.6 Determination of Heavy Metals

Heavy metals in cheese samples were determined using inductively coupled plasma optical emission spectroscopy (ICP‐OES) (Thermo Scientific iCAP PRO X ICP‐OES, Massachusetts, USA). 1 g of homogenized cheese samples was weighed and placed in tubes prepared for this purpose. Then, 4 mL of 65% (v/v) nitric acid and 2 mL of 30% (v/v) hydrogen peroxide were added using a pipette and placed in a microwave. The samples were microwaved at 90°C, 170°C, and 210°C for 8 min, 10 min, and 25 min, respectively. The samples were cooled after the microwaving process and transferred. The samples were transferred to sterile tubes and diluted with ultrapure water. Heavy metals were determined using an ICP‐OES device (Altun and Aydemir 2020).

### 2.7 Extraction and Detection of Aflatoxin in Cheese Samples

Aflatoxin was extracted from cheese samples according to Ewida et al. (2024). Weighing 1 g of the crushed and ground sample and mixing it with 4 mL of 100% pure methanol, stirring it vigorously using a hand mixer (vortex) for 5 min. Then, the mixture was centrifuged at 4000 rpm for 10 min. After centrifugation, 1 mL of the filtrate was transferred, and then the filtrate was concentrated using a rotary evaporator at 70°C. The concentrated filtrate was then mixed with 800 μL of 1× PBS solution and stirred with a vortex for 1 min. The test was conducted in the laboratories of the Scientific Research Authority—Environment and Water Department using a German‐made high‐performance liquid chromatography (SYKAM) device. The carrier phase was acetonitrile: distilled water (70:30). A C18‐ODS (25 cm × 4.6 mm) separation column was used to separate mycotoxins. A fluorescence detector (ex = 365 nm, em = 445 nm) was used to detect mycotoxins. The carrier phase flow rate was 0.7 mL/min. 10 μL were injected into the HPLC.

### 2.8 Statistical Analyses

SPSS 26.0 software was used for one‐way ANOVA, and the differences were analyzed for significance using Duncan's multiple comparisons at *p* < 0.05. All analytical methods were done in three replications for each sample. Calibration standards and certified reference materials were applied for instrumental analysis techniques. Each instrument was calibrated before use, and blank samples were run to detect any potential contamination.

## 3 Results and Discussion

### 3.1 Determination of Natamycin

**FIGURE 2:**
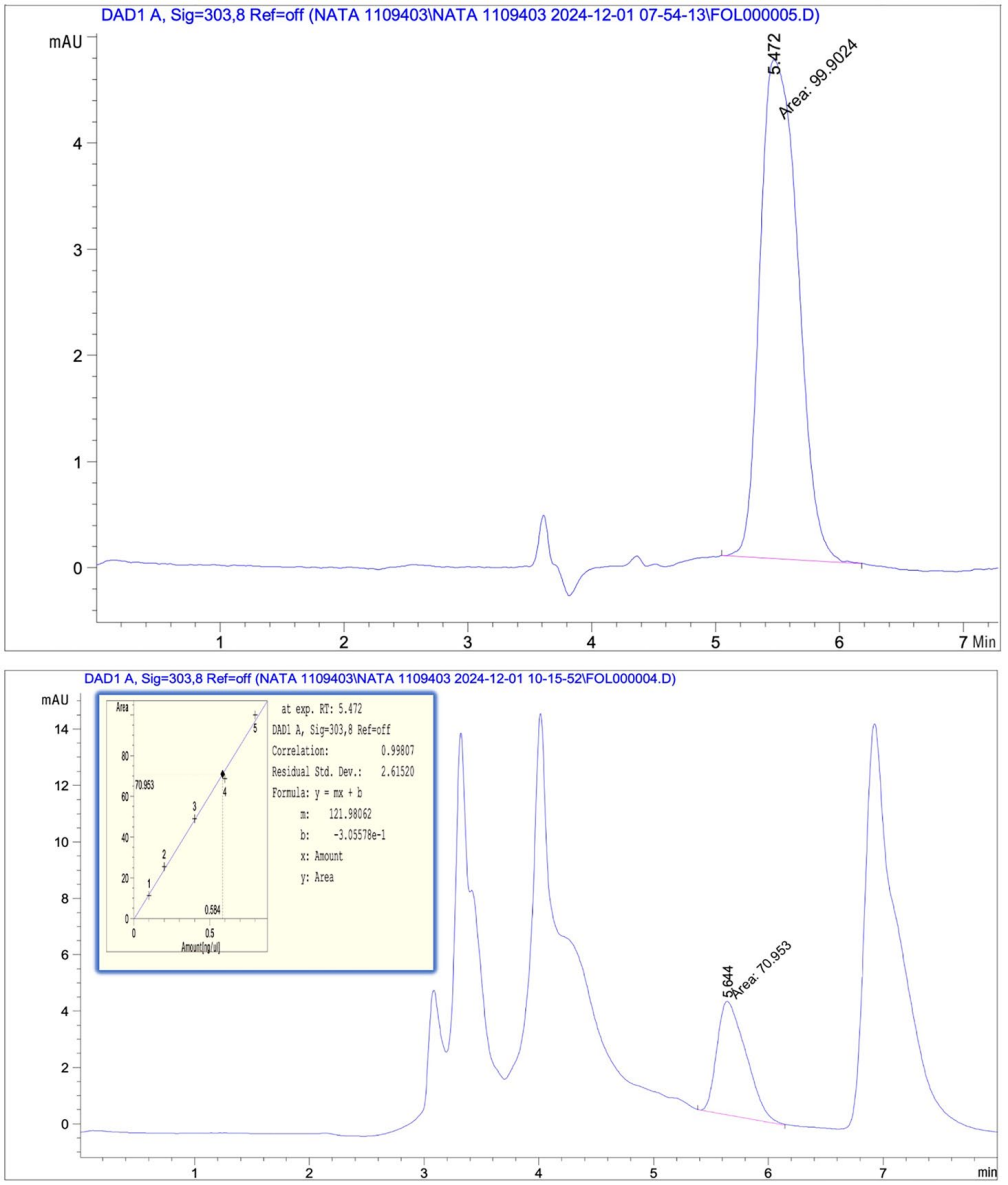
HPLC profile of standard natamycin (up), HPLC profile of natamycin in a sample of Saudi soft cheese (down).

The results showed the absence of natamycin in all cheeses imported from Iran, Saudi Arabia, and Turkey, except for the CHS2‐S sample. The natamycin preservative content in Saudi soft cheese was 0.584 ppm, as shown in the HPLC profile of the natamycin standard and the HPLC profile of the CHS2‐S soft cheese sample (Figure 2). The natamycin peak appeared at a retention time of 5.472 min for the standard and 5.644 min for the CHS2‐S Saudi soft cheese sample. The correlation coefficient of the calibration curve indicates a strong linear relationship between standard concentrations and analysis readings. In this study, the correlation coefficient of the calibration curve was higher than 0.998, indicating a high consistency with the straight line, thus enhancing the accuracy of the results in determining the concentration of the target substance in the samples.

These findings align with Zamani Mazdeh et al. (2017), who analyzed 195 samples from 15 brands and 5 types of commercial cheese and yogurt in Tehran, Iran. The natamycin content ranged from 0.132 to 1.508 mg/kg, with an average of 0.717 mg/kg. The results were also consistent with the Turkish standard, which states that fermented dairy products should not contain any preservatives; although some producers add natamycin as a preservative to prevent the growth of yeast and mold (Bilgic Alkaya and Karalomlu 2016).

### 3.2 Determination of Nisin

**FIGURE 3:**
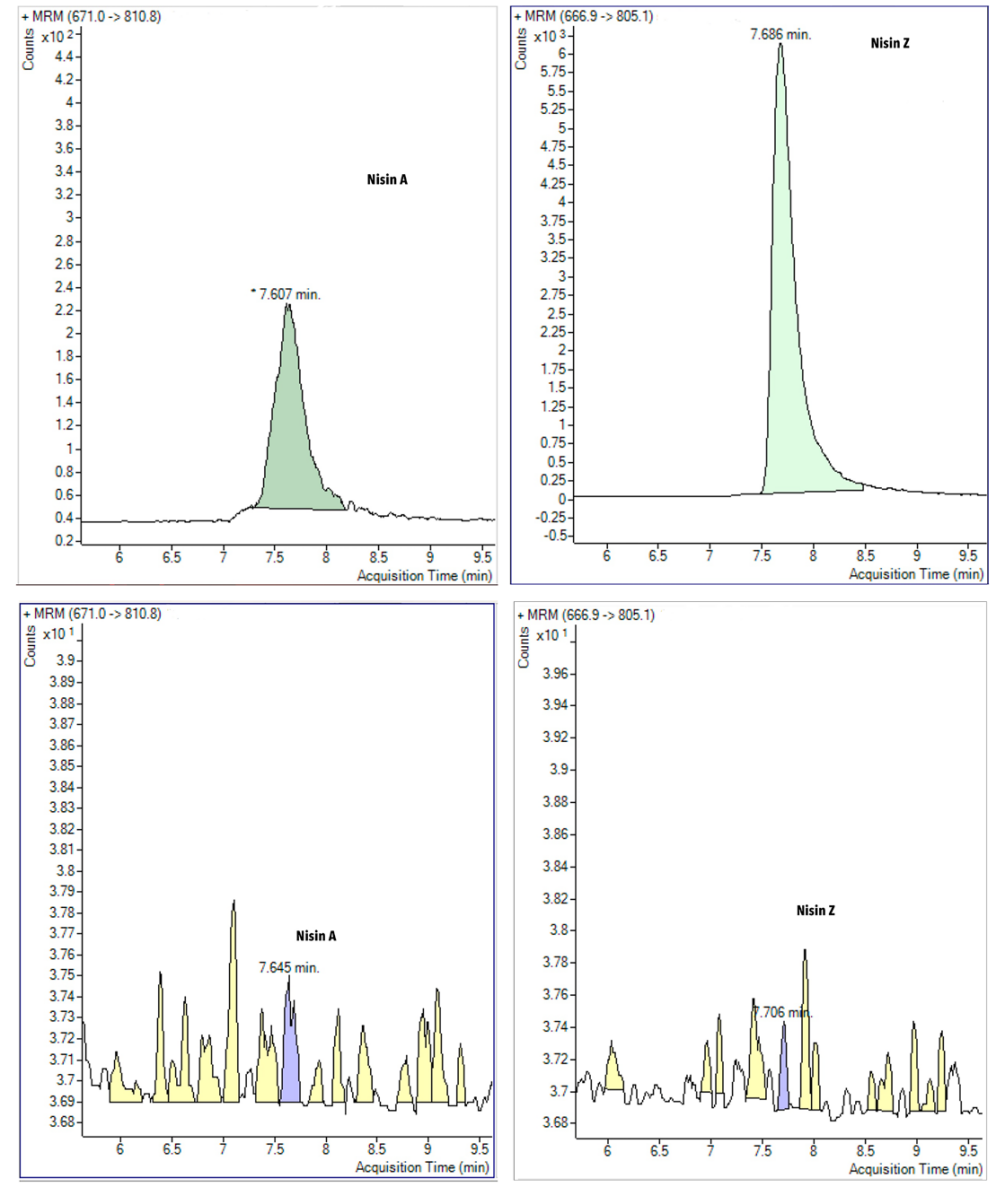
LC–MS–MS profile of standard nisin A and Z (up), nisin A in CHH1‐T and nisin Z in CHH2‐S (down).

Some countries prefer Nisin Z over Nisin A as a natural preservative (Zhejiang Silver‐Elephant Bio‐Engineering, China). Therefore, the simultaneous quantification of nisin A and nisin Z in foods is very important (Ko et al. 2015). LC–MS–MS analysis was performed to simultaneously measure nisin A and nisin Z in cheese samples. The primary ions of the standard nisin A and Z were 671.0 and 666.9 m/z at retention times of 7.607 and 7.686 min, respectively, while the dissociation ions of nisin A were 810.8 and 805.1 m/z for nisin Z (Figure 3).

The results showed that nisin A and Z were not detected in any of the soft or semi‐hard cheese samples. In contrast, the amount of nisin A in CHH1‐T was 15.6 ± 10.3 ng/mL and the amount of nisin Z in CHH2‐S was 80.0 ± 11.2 ng/mL (Figure 3). This result was lower than the limits recommended by both the European Commission Regulation and CODEX STAN 283–1978 2013, which recommend that the maximum level of nisin (E234) should not exceed 12.5 ppm in matured cheese, processed cheese, and cream.

Nisin can only be used in processed cheese at concentrations below the maximum level of 250 ppm according to the Food Additives Regulation (Ko et al. 2016) in Korea. However, nisin is permitted in various types of cheese products, not just processed cheese, in many countries, including the European Union and the United States. The results are consistent with those of Ko et al. (2015), who examined 36 types of natural cheese imported from the Netherlands for the amounts of nisin A and nisin Z. Neither nisin A nor nisin Z was detected in fresh cheese, soft cheese, semi‐hard cheese, and hard cheese. However, the results are inconsistent with those of Schneider et al. (2011), who found that nisin Z levels in cheese samples were (1.6–1.7) ppm. Therefore, we could not confirm whether nisin was intentionally added to the cheese as a preservative or whether it was produced naturally during cheese fermentation (EC 2008). Further studies are therefore needed on the expected amounts of nisin that could be formed naturally during cheese manufacturing or fermentation, to determine whether nisin was an artificial additive or a natural product in the cheese sample.

### 3.3 Fatty Acids Determination and Lipid Quality Indices

**FIGURE 4:**
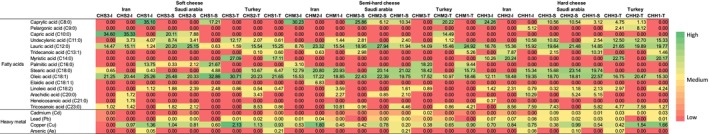
Heat‐map of fatty acid composition and heavy metal contaminations in cheeses categorized by country and cheese type.

Figure 4 shows a heatmap illustrating the relative abundance of key fatty acids in cheese samples, determined by gas chromatography–mass spectrometry (GC–MS), with green indicating the highest concentrations and red the lowest. Table 2 also shows the percentages of saturated fatty acids (SFA) and unsaturated fatty acids (UFA) in cheeses from Iran, Saudi Arabia, and Turkey. For soft cheeses, the percentage of SFA ranged between 50.7398% and 59.0935% in Iranian samples, 40.6842% and 51.2022% in Saudi samples, and 40.3419% and 40.8827% in Turkish samples. As for semi‐hard cheeses, the percentage of SFA ranged between 46.7484% and 54.8778% in Iranian samples, 51.559% and 55.0496% in Saudi samples, and 50.7052% and 59.5623% in Turkish samples. While in hard cheeses, SFA percentage ranged from 51.6622% to 64.8403% in Iranian samples, from 60.396% to 66.2744% in Saudi samples, and from 49.4273% to 68.8137% in Turkish samples. The results showed that the predominant saturated fatty acids in the samples analyzed in this study were Lauric acid (C12:0), Palmitic acid (C16:0), and Stearic acid (C18:0). Moreover, the results showed the presence of Arachidic acid (C20:0) in some cheese samples, which is not usually found in milk fat and is present in vegetable oils such as palm oil.

**TABLE 2:** Lipid quality indices in cheese samples using gas chromatography–mass spectrometry.

	Iran			Saudi Arabia			Turkey		
	CHS3‐I	CHS2‐I	CHS1‐I	CHS3‐S	CHS2‐S	CHS1‐S	CHS3‐T	CHS2‐T	CHS1‐T
Soft cheese									
SFA %	50.73 ± 0.98^c^	59.09 ± 1.43^a^	54.58 ± 0.53^b^	51.20 ± 1.22^c^	40.68 ± 1.42^e^	44.38 ± 0.47^d^	40.84 ± 0.43^e^	40.34 ± 1.19^e^	40.88 ± 1.27^e^
UFA %	21.25 ± 0.18^d^	20.44 ± 0.29^d^	26.38 ± 0.54^c^	27.35 ± 0.98^c^	22.72 ± 0.41^d^	35.34 ± 1.49^a^	31.57 ± 1.06^b^	20.76 ± 0.79^d^	22.11 ± 0.75^d^
DHFA %	21.90 ± 0.09^d^	20.44 ± 1.29^d^	26.81 ± 0.79^c^	27.35 ± 0.98^c^	22.72 ± 0.41^d^	40.22 ± 1.05^a^	31.57 ± 1.06^b^	31.43 ± 0.77^b^	28.58 ± 1.46^c^
HSFA %	50.09 ± 1.07^c^	59.09 ± 1.13^a^	54.15 ± 1.28^b^	51.20 ± 1.22^c^	40.68 ± 1.42^d^	39.50 ± 0.91^d^	40.84 ± 1.43^d^	29.67 ± 1.21^f^	34.41 ± 1.56^e^
Hp_o_/Hp_er_	1.46 ± 0.83^a^	1.35 ± 0.26^ab^	1.76 ± 0.09^a^	1.33 ± 0.24^ab^	0.83 ± 0.32^b^	1.58 ± 0.50^a^	1.10 ± 0.09^b^	1.33 ± 0.60^ab^	0.68 ± 0.36^b^
Semi‐hard cheese									
SFA %	54.87 ± 0.78^b^	46.74 ± 0.84^d^	50.99 ± 0.95^c^	51.55 ± 0.90^bc^	55.04 ± 0.96^b^	52.27 ± 0.43^bc^	50.70 ± 1.52^c^	52.17 ± 1.02^bc^	59.56 ± 1.23^a^
UFA %	15.53 ± 1.33^d^	24.05 ± 0.90^a^	22.43 ± 0.84^ab^	22.42 ± 0.63^ab^	22.38 ± 0.50^ab^	21.35 ± 0.75^b^	18.40 ± 0.58^c^	10.96 ± 0.54^e^	18.45 ± 0.67^c^
DHFA %	28.32 ± 1.05^d^	44.86 ± 0.21^a^	43.37 ± 0.31^b^	22.42 ± 0.63^e^	42.52 ± 1.21^b^	42.37 ± 0.79^b^	36.82 ± 0.36^c^	10.96 ± 0.54^f^	34.19 ± 1.12^d^
HSFA %	42.09 ± 1.06^b^	25.94 ± 0.53^d^	30.06 ± 0.48^c^	51.55 ± 0.90^a^	34.91 ± 1.25^c^	31.25 ± 1.39^c^	32.28 ± 1.74^c^	52.17 ± 1.02^a^	43.82 ± 0.78^b^
Hp_o_/Hp_er_	1.30 ± 0.17^a^	0.73 ± 0.06^ab^	1.44 ± 0.38^a^	1.18 ± 0.36^a^	0.80 ± 0.11^ab^	1.78 ± 0.83^a^	0.57 ± 0.10^b^	0.70 ± 0.19^ab^	0.53 ± 0.17^b^
Hard cheese									
SFA %	51.66 ± 1.22^e^	52.02 ± 1.43^e^	64.84 ± 1.03^b^	66.27 ± 1.44^a^	60.39 ± 1.60^c^	61.53 ± 1.06^c^	68.81 ± 1.37^a^	49.42 ± 0.73^e^	58.00 ± 0.75^d^
UFA %	13.53 ± 0.12^c^	20.78 ± 0.24^b^	20.13 ± 0.75^b^	19.01 ± 0.84^b^	19.98 ± 0.47^b^	24.70 ± 0.73^a^	19.72 ± 0.80^b^	20.46 ± 0.59^b^	19.54 ± 0.61^b^
DHFA %	13.53 ± 0.21^e^	20.78 ± 0.24^c^	35.48 ± 1.03^b^	34.70 ± 1.14^b^	43.12 ± 0.51^a^	44.44 ± 1.35^a^	19.72 ± 0.80^cd^	17.46 ± 1.59^d^	19.54 ± 1.61^cd^
HSFA %	51.66 ± 1.22^cd^	52.02 ± 1.43^cd^	49.49 ± 0.75^d^	50.59 ± 1.28^d^	37.25 ± 0.56^f^	41.79 ± 0.44^e^	68.81 ± 1.37^a^	53.42 ± 0.73^c^	64.00 ± 0.75^b^
Hp_o_/Hp_er_	0.50 ± 0.05^a^	0.58 ± 0.38^a^	0.78 ± 0.51^a^	0.96 ± 0.85^a^	0.93 ± 0.02^a^	1.66 ± 0.41^a^	0.44 ± 0.03^a^	1.02 ± 0.88^a^	0.48 ± 0.03^a^

*Note:* Different letters within the same row indicate significant differences (*p* < 0.05).

The results in Figure 4 and Table 2 showed that the percentage of UFA in soft cheese samples ranged between 20.4429% and 26.3854%, 22.7241% and 35.3449%, and 20.7679% and 31.5706% for Iranian, Saudi, and Turkish samples, respectively. The results showed that the percentage of UFA in semi‐hard cheese samples ranged between 15.5333% and 24.059%, 21.3575% and 22.4263%, and 10.9654% and 18.4567% for Iranian, Saudi, and Turkish samples, respectively. The results also showed that the percentage of UFA in hard cheese samples ranged between 13.53% and 20.7824%, 19.0184% and 24.7073%, and 19.5461% and 20.4659% for Iranian, Saudi, and Turkish samples, respectively. The results showed that (C18:1) oleic acid and (C18:2) linoleic acid were the most abundant unsaturated fatty acids in the cheese samples.

The majority of unsaturated fatty acids in the human diet are found in the cis form. However, trans fatty acids (TFAs) can be found in the foods we eat. Companies are no longer allowed to produce foods containing trans fats, according to regulations set by both the World Health Organization (WHO) and the U.S. Food and Drug Administration (FDA). However, some of these products may contain trace amounts of industrially produced trans fats (Bhandari et al. 2020). The results showed that the trans fatty acid elaidic acid (C18:1n9, t) was only detected in sample CHM2‐I, with an average value of 6.8345%. This result was inconsistent with that reported by Pipoyan et al. (2021). The Food and Agriculture Organization (FAO) and the World Health Organization (WHO) recommended in 1994 that fats intended for human consumption should contain less than 4% total trans fats. Many factors can influence the trans fat content of cheese, including geographical factors. Furthermore, heat treatment during cheese manufacturing can affect the trans fat content. Pasteurization or microwave heating of milk has been shown to increase trans fatty acids (Herzallah et al. 2005). Paszczyk and Łuczyńska (2020) suggested that high levels of C18:1 trans in cheese may be due to different feeding regimes, as cheese made from milk obtained from animals fed fodder with added oilseeds or fresh grass may contain more of these isomers.

The average values of SFA, UFA, DHFA, and HSFA for soft cheese samples produced in Iran, Saudi Arabia, and Turkey, respectively. The results of the statistical analysis showed that the average SFA values for Iranian soft cheese samples were significantly higher than those for Turkish soft cheese samples, while there were no significant differences between the Iranian and Saudi samples and the Saudi and Turkish cheese samples. The average UFA value in Saudi soft cheese was significantly higher than that of Iranian soft cheese, while there were no significant differences between the Saudi and Turkish samples and the Iranian and Turkish cheese samples. The average DHFA value in cheeses manufactured in Saudi Arabia was not significantly different from that in Turkish cheeses, while the average DHFA value was significantly higher for both Saudi and Turkish samples compared to the Iranian samples. The results of statistical analysis of the average values of HSFA also showed significant differences between all types of cheese manufactured in Iran, Saudi Arabia, and Turkey.

The average values of SFA, UFA, DHFA, and HSFA for semi‐solid cheese samples manufactured in Iran, Saudi Arabia, and Turkey, respectively. The results of the statistical analysis of the average SFA values showed no significant differences between all types of cheese manufactured in Iran, Saudi Arabia, and Turkey. The average UFA value in Saudi semi‐solid cheese was significantly higher than that of Turkish cheese. However, there were no significant differences between the Saudi and Iranian samples on the one hand, and the Iranian and Turkish cheese samples on the other. The results of the statistical analysis also indicated that the average DHFA value in cheese manufactured in Iran differed significantly from that in Turkish cheese, while there were no significant differences when comparing the average DHFA values for the Iranian and Saudi samples on the one hand, and the Saudi and Turkish samples on the other. The results of the statistical analysis of the average HSFA values also showed significant differences between cheese manufactured in Turkey and Iran, while there were no significant differences when comparing the average values of HSFA for both the Turkish and Saudi samples on the one hand and the Iranian and Saudi samples on the other hand.

The average values of SFA, UFA, DHFA, and HSFA for Iranian, Saudi, and Turkish hard cheese samples, respectively. The results of the statistical analysis of the average SFA values showed significant differences between cheeses manufactured in Saudi Arabia and Iran, while there were no significant differences between the Saudi and Turkish samples on the one hand and the Iranian and Turkish cheese samples on the other. The average UFA values did not differ significantly among all types of cheese manufactured in Iran, Saudi Arabia, and Turkey. The results of the statistical analysis also showed that the average DHFA value in cheese manufactured in Saudi Arabia differed significantly from both Turkish and Iranian cheeses, while there were no significant differences when comparing the average DHFA values for the Turkish and Iranian samples. The results of the statistical analysis of the average HSFA values also showed significant differences between cheeses manufactured in Turkey and those manufactured in Saudi Arabia and Iran. However, there were no significant differences when comparing the average HSFA values of the Iranian and Saudi samples.

These results were inconsistent with what was found by Ali et al. (2024) who indicated that the percentage of SFA was (72.32% ± 0.10%), (68.89% ± 0.35%), (72.47% ± 0.29%) in each of the Ras Cheese, Cheddar Cheese, and Gouda Cheese samples, respectively. Donmez et al. (2005) confirmed that the percentage of SFA in hard cheeses ranged between 69.70% and 75.76% when studying some traditional Turkish cheeses. The results also did not agree with the findings of Paszczyk et al. (2022), who showed that the percentage of unsaturated fatty acids in cheese made from cow's milk during the summer and winter seasons was (30.93% ± 0.94%) and (28.50% ± 0.47%), respectively. The study showed that the percentages of DHFA% and HSFA% differed significantly between soft, semi‐hard, and hard cheeses produced in Iran, Saudi Arabia, and Turkey, highlighting the effect of both the cheese type and the producing country on the lipid composition. Saudi hard cheeses achieved the highest percentage of DHFA%. The results of this study are consistent with Margalho et al. (2021), who explained that the levels of DHFA and HSFA depend on the content of monounsaturated fatty acids and fatty acids. Polyunsaturated fatty acids, stearic acid, and medium‐chain saturated fatty acids, respectively, are important. Higher levels of monounsaturated fatty acids and polyunsaturated fatty acids impact health indicators, so cheese quality can be improved by improving animal diets and manufacturing techniques. These findings support the importance of monitoring fatty acid levels to enhance food product quality and improve public health.

The Hpo/Hper ratio is related to the functional activity of FFA, the metabolism of lipoproteins for cholesterol transport in plasma, and the development of cardiovascular disease risk. The Hpo/Hper ratio is a potential index of the probable impact of dietary fat on cardiovascular health. High ratios suggest a favorable balance of fatty acids that will decrease the cholesterol level, a suggestion for dietary application in reducing the risk of heart disease. The variations of Hpo/Hper ratios between the cheeses reflect variations in possible health impacts based on their fatty acid contents. The values of Hpo/Hper in soft cheeses ranged between (1.3526–1.7609) in Iranian samples, (0.8332–1.5850) in Saudi samples, and (0.6836–1.3360) in Turkish samples. As for semi‐hard cheeses, the values of Hpo/Hper ranged between (0.7386–1.4438) in Iranian samples, (0.8011–1.7883) in Saudi samples, and (0.5371–0.7091) in Turkish samples. While in hard cheeses, it showed that the values of Hpo/Hper ranged between (0.5005–0.7851) in Iranian samples, and (0.9302–1.6641) in Saudi samples, and (0.4443–1.0288) in Turkish samples. The results obtained were consistent with what Paszczyk and Łuczyńska (2020) indicated, as they explained that the higher this ratio, the more desirable it is from a health perspective.

### 3.4 Determination of Heavy Metals

Figure 4 shows the heavy metals content in cheese from Iran, Saudi Arabia, and Turkey. Average heavy metal values were cadmium (Cd) at 0.0014 ± 0.0001 mg/kg, lead (Pb) at 0.0061 ± 0.0010 mg/kg, copper (Cu) at 1.097 ± 0.204 mg/kg, and arsenic (As) at 0.051 ± 0.020 mg/kg in soft cheeses, while they were (0.0053 ± 0.0020), (0.0072 ± 0.0020), (0.014 ± 0.678), (0.023 ± 0.002), respectively, in semi‐hard cheeses, and (0.025 ± 0.011), (0.035 ± 0.015), (0.869 ± 0.175), (0.028 ± 0.017), respectively, in hard cheeses. It was observed that the highest values of Cd, Pb, Cu, and As were in the samples CHH1‐S, CHH3‐I, CHM2‐I, and CHS2‐T, respectively.

**FIGURE 5:**
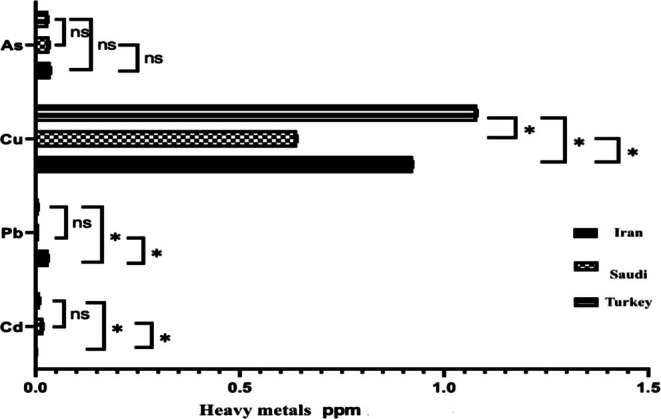
Comparison of average mineral values in cheeses produced in Iran, Saudi Arabia, and Turkey.

The statistical analysis showed significant effects for “cheese type” and “country of production”, as well as a significant interaction between them. Figure 5 compares average mineral values of cheeses from Iran, Saudi Arabia, and Turkey. Cd values differed significantly (*p* < 0.05) between Iranian cheese and those from Saudi Arabia and Turkey, but no significant difference (*p* > 0.05) was found between Saudi Arabian and Turkish cheeses. Lead (Pb) levels differed significantly (*p* < 0.05) between Iranian cheeses and those from Saudi Arabia and Turkey. No significant difference (*p* > 0.05) was found between Saudi Arabian and Turkish cheeses. Copper (Cu) levels also showed significant differences (*p* < 0.05) among Iranian, Saudi Arabian, and Turkish cheeses. Arsenic (As) levels, however, showed no significant differences (*p* > 0.05) among the three countries (Figure 5).

The cadmium (Cd) values obtained from the samples analyzed in the current study were in line with the permissible limit for cheese set by the European Union EC (2006) and Codex Alimentarius, with the exception of the CHH3‐S cheese sample, which exhibited Cd levels exceeding the global average of 0.05 mg/kg (Anguebes‐Franseschi et al. 2023). These findings align with those of Ibrahim (2004), who reported that 40%–90% of toxic heavy metals present in milk remain in cheese after processing, especially in hard cheeses compared to soft cheeses. In addition, there is a potential health risk associated with using salt contaminated by heavy metals in cheese production.

Lead (Pb) levels in most imported cheese samples from Iraqi markets in Babylon Governorate were below the global average of 0.02 mg/kg, according to European Union (European Commission (EU) 2006) and Codex standards. However, samples CHM2‐I, CHH3‐I, CHH2‐I, CHH1‐I, CHH1‐S, CHH3‐S, and CHH3‐T exceeded this limit (Muneam and Abojassim 2023). The Pb values in the study aligned with Turkish specification of 0.02–2 mg/kg (Çetinkaya et al. 2016). Al Sidawi et al. (2021) suggested that lead's affinity for casein, which concentrates in the curd during cheese‐making, contributes to its presence in cheese. Dağcilar and Gezer (2021) proposed that lead contamination could also result from milk transport or storage in lead‐coated containers. Furthermore, Anguebes‐Franseschi linked lead in cheese to contaminated alfalfa used as cattle feed (Anguebes‐Franseschi et al. 2023).

The results showed that Cu values in foodstuffs vary depending on environmental conditions, cooking methods, processing, and food handling (Christophoridis et al. 2019). Notably, some studies in Iran, where many mining areas are located, have shown very high levels of copper in the analyzed samples. In the current study, the average Cu values ranged from (0.196 ± 0.09) to (1.802 ± 0.482) mg/kg. When comparing these results with measurements conducted in other countries, it can be noted that the detected concentration levels are lower than those recorded in previous studies, where Cu values reached 3250 μg/kg in feta cheese in Egypt, 300–1600 μg/kg in Kasar cheese in Ankara, 640–870 μg/kg in white cheese in Turkey, and 300–850 μg/kg in Spain (Christophoridis et al. 2019). Furthermore, the Cu values were also beneath the permissible limits set by Georgian regulation (1.26–2.46 mg/kg) but exceeded the maximum permissible limits established by the Codex Alimentarius Commission and the International Dairy Federation (10 μg/kg) (Al Sidawi et al. 2021; Ghafari and Sobhanardakani 2017).

The results showed that, as values in all examined cheese samples were lower than the global average of 1.0 mg/kg according to the Codex Alimentarius Commission, except cheese samples CHS1‐I, CHH3‐S, CHS3‐T, CHS2‐T, CHM2‐I, CHH2‐T, and CHH1‐T, which showed values higher than the global average (Çetinkaya et al. 2016). The heavy metal content in cheese varies due to many factors, such as manufacturing processes and the potential contamination of equipment during production, packaging, and storage. Therefore, it is necessary to control the manufacturing process at every step to identify the source and levels of contamination and ensure the required product quality (Abd‐El‐Rahim et al. 2012). In short, animal feeding, contaminated water sources, or cheesemaking equipment can be a source of heavy metal contamination. Differences in agricultural practices and soil/environmental contamination can also be a source of heavy metals in dairy products. Control measures like the Codex Alimentarius Commission and the European Union should be kept under close observation to maintain the integrity of consumers' safety.

### 3.5 Detection of Aflatoxin M1

**FIGURE 6:**
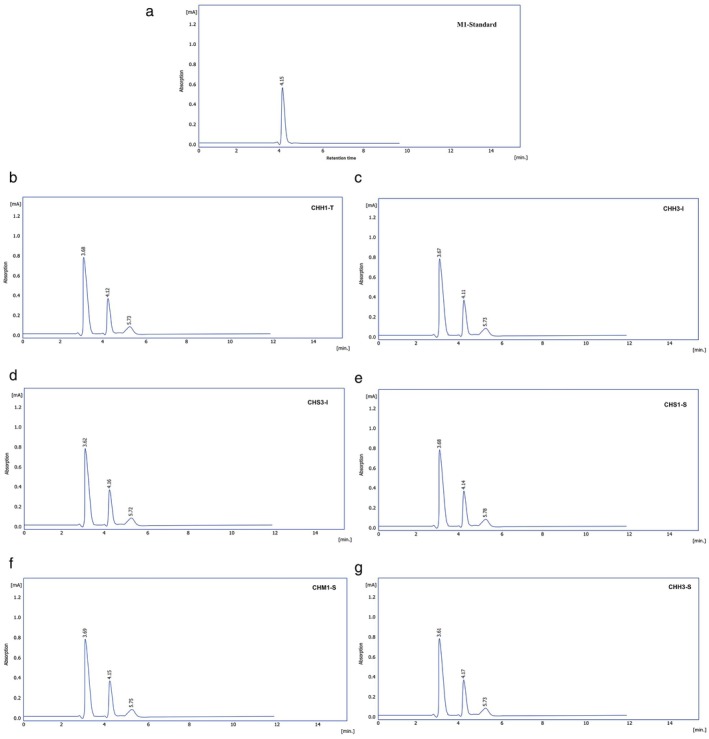
HPLC chromatograms of standard M1, CHH3‐I, CHM1‐S, CHS1‐S, CHS3‐I, CHH3‐S, and CHH1‐T.

Figure 6 shows the HPLC profile of standard aflatoxin M1 and the HPLC profiles of aflatoxin M1 content in the cheese samples used in the study, namely CHS3‐I, CHS1‐S, CHM1‐S, CHH3‐I, CHH3‐S, and CHH1‐T, which were produced in Iran, Saudi Arabia, and Turkey. Table 3 shows aflatoxin M1 detected in two samples (7.7%) out of a total of 27 soft cheese samples, one sample (3.7%) out of a total of 27 semi‐hard cheese samples, and three samples (11.11%) out of a total of 27 hard cheese samples. All positive samples exceeded the maximum limit of 0.05 ppb set by the European Union and Turkey (Turkish Food Codex, TFC) and some other countries (Ashraf 2012). Škrbić et al. (2015) indicated that the M1 concentration was 2.3–3.3 times higher in soft cheeses and 3.9–5.8 times higher in hard cheeses compared to the milk from which the cheese was made. The results obtained are consistent with Mudannayake et al. (2024) who explained that hard cheese products with a higher casein content and lower water content had a higher M1 concentration compared to other cheese types. Škrbić et al. (2015) also confirmed that animal feed contaminated with aflatoxin‐causing molds and stored under inappropriate conditions can also lead to elevated M1 concentrations in milk and cheese. Exceedances of the acceptable maximum level for aflatoxin M1 are of significant concern due to the compound's established carcinogenicity. Chronic exposure to aflatoxin M1 can cause severe health impacts, such as liver cancer and immune suppression. Therefore, it is necessary to implement strict control on animal feed and milk production to avoid risks of contamination. Exceeding regulatory limits for aflatoxins and heavy metals in foods may have serious legal and commercial concerns. Foods that fail to meet international standards or national food safety controls can be subject to import bans, recalls, fines, or damage to brand popularity. Moreover, the presence of non‐compliant levels of contaminants may corrupt consumer trust and pose public health risks, necessitating stricter import inspection and producer accountability.

**TABLE 3:** Estimation of aflatoxin M1 in cheese samples.

Type of cheese/country of manufacture		Retention time	Area	Concentration (ppb)	Positive samples/total samples
M1 standard		4.15	524.90	5	
Soft cheese	CHS3‐I	4.16	2124.58	20.237 ± 1.245^c^	(7.70%) 2/27
	CHS1‐S	4.14	2365.07	22.528 ± 1.041^bc^	
Semi‐hard cheese	CHM1‐S	4.15	2365.99	22.537 ± 1.607^bc^	(3.70%) 1/27
Hard cheese	CHH3‐I	4.11	2965.08	28.244 ± 1.131^a^	(11.11%) 3/27
	CHH3‐S	4.17	2632.50	25.076 ± 1.468^b^	
	CHH1‐T	4.12	2560.22	24.387 ± 1.141^b^	
Total positive samples/total samples					(7.40%) 6/81

*Note:* Different letters within the same column indicate significant differences (*p* < 0.05).

### 3.6 Risk Assessment

Using the mean contaminants concentrations determined in the present study, a chronic daily intake (CDI) was calculated using an average cheese consumption (30 g/day/person), a mean body weight of 70 kg, and 30 years of exposure. Aflatoxin M1 in positive samples was also compared against the tolerable daily intake (TDI) levels advised by international health organizations. Hazard quotient values were obtained using the equation: $\mathrm{HQ}=\frac{\mathrm{CDI}}{\mathrm{RfD}/\mathrm{TDI}}$. The oral reference doses (RfD) were taken from international health agencies (Cd: 0.0018, Pb: 0.0036, Cu: 0.04 mg/kg/day; aflatoxin M1: 2 ng/kg/day).

It is noted that all heavy metals had HQ < 1, representing no immediate non‐carcinogenic health risk from the consumption of cheese with positive heavy metals, but the HQ values calculated for some samples of cheese, especially for aflatoxin M1, were concerning. Six aflatoxin‐positive samples had HQ > 1, with some values exceeding five‐fold. These findings suggest a chronic exposure risk, particularly for regular and frequent consumption. It is stated that when HQ is < 1, it indicates tolerable exposure, while > 1 indicates an intolerable level of exposure. The results of the risk assessment are tabulated in Table 4.

**TABLE 4:** Estimated CDI and HQ values for heavy metals and aflatoxin M1 in positive cheese samples.

Sample ID	Contaminant	C (mg/kg)	CDI (mg/kg/day)	RfD TDI mg/kg/day)	HQ	Risk level
CHH1‐S	Cd	0.09	1.66 × 10^−5^	0.0018	0.0092	No risk
CHM2‐I	Pb	0.04	7.36 × 10^−6^	0.0036	0.0020	No risk
CHH3‐I	Pb	0.07	1.29 × 10^−5^	0.0036	0.0036	No risk
CHH2‐I	Pb	0.07	1.29 × 10^−5^	0.0036	0.0036	No risk
CHH1‐I	Pb	0.08	1.47 × 10^−5^	0.0036	0.0041	No risk
CHH1‐S	Pb	0.03	5.52 × 10^−6^	0.0036	0.0015	No risk
CHH3‐S	Pb	0.03	5.52 × 10^−6^	0.0036	0.0015	No risk
CHH3‐T	Pb	0.03	5.52 × 10^−6^	0.0036	0.0015	No risk
CHS1‐I	Cu	1.36	2.50 × 10^−4^	0.04	0.0063	No risk
CHH3‐S	Cu	1.04	1.91 × 10^−4^	0.04	0.0048	No risk
CHS3‐T	Cu	2.13	3.92 × 10^−4^	0.04	0.0098	No risk
CHS2‐T	Cu	1.13	2.08 × 10^−4^	0.04	0.0052	No risk
CHM2‐I	Cu	1.80	3.31 × 10^−4^	0.04	0.0083	No risk
CHH2‐T	Cu	1.54	2.83 × 10^−4^	0.04	0.0071	No risk
CHH1‐T	Cu	1.66	3.05 × 10^−4^	0.04	0.0076	No risk
CHS3‐I	Aflatoxin M1	2.0237 × 10^−5^	8.67 × 10^−7^	2.0 × 10^−7^	4.34	High risk
CHS1‐S	Aflatoxin M1	2.2528 × 10^−5^	9.66 × 10^−7^	2.0 × 10^−7^	4.83	High risk
CHM1‐S	Aflatoxin M1	2.2537 × 10^−5^	9.66 × 10^−7^	2.0 × 10^−7^	4.83	High risk
CHH3‐I	Aflatoxin M1	2.8244 × 10^−5^	1.21 × 10^−6^	2.0 × 10^−7^	6.03	High risk
CHH3‐S	Aflatoxin M1	2.5076 × 10^−5^	1.07 × 10^−6^	2.0 × 10^−7^	5.37	High risk
CHH1‐T	Aflatoxin M1	2.4387 × 10^−5^	1.05 × 10^−6^	2.0 × 10^−7^	5.26	High risk

The findings of the current study necessitate continual vigilance and public awareness aimed at minimizing exposure. Therefore, we recommend that manufacturers exert stricter quality control programs, including regular testing of their raw materials and products for heavy metals and mycotoxins; control organizations scale up import screening exercises for dairy products and enforce international safety standards on our imported dairy products; consumers purchase cheese brands with proper labeling and certified safety testing; and lastly, public awareness campaigns about the health risks associated with ingesting contaminated dairy products.

These findings imply that there is an urgent need for (1) stricter controls on milk sourcing and cheese processing; (2) routine testing for aflatoxin monitoring in the dairy supply chains; (3) guidance to consumers toward safer and certified consumer products; and (4) regulatory enforcement on maximum residue limits for aflatoxin in cheese products.

## 4 Conclusion

This study shows the distinct effect of geographical origin and processing methods on the safety and quality of imported cheeses in Babylon Governorate. Variations in the levels of preservatives used, especially the detection of nisin and the limited use of natamycin, showed compliance in some cases and deviations from food safety standards in others. This raises concerns about potential health risks for consumers. In addition, the analysis of lipid profiles, which showed both high levels of saturated fatty acids and significant diversity, indicated indicators of the fat quality of the cheese samples, confirming that these variations could be due to the origin and production methods. The detection of heavy metals such as cadmium and lead in some cheese samples, even in some cases at levels above the permissible limits, indicates the urgent need for further monitoring to protect public health. The detection of aflatoxin M1 in several samples also added to food safety concerns and underscored the need for careful feed management to prevent this contamination. The results of this study suggest that regulatory agencies need to implement more stringent quality assurance measures and provide public education on the safe consumption of cheese. Given the long‐term effects of these contaminants on public health, extensive monitoring and research are essential to establish and develop effective programs that ensure consumer safety and improve the overall quality of imported dairy products. This study is an important step in promoting better standards and practices in the dairy industry.

## Author Contributions

**Mohammed A. Falih:** data curation (equal), formal analysis (equal), investigation (equal), writing – original draft (equal). **Qausar Hamed Alkaisy:** conceptualization (equal), data curation (equal), formal analysis (equal), methodology (equal), validation (equal), writing – original draft (equal). **Ammar B. Altemimi:** conceptualization (equal), data curation (equal), project administration (equal), supervision (equal), validation (equal), writing – review and editing (equal). **Tarek Gamal Abedelmaksoud:** data curation (equal), methodology (equal), validation (equal), writing – review and editing (equal).

## Conflicts of Interest

The authors declare no conflicts of interest.

## Data Availability

The data that support the findings of this study are available on request from the corresponding author.
